# Potential MRI Biomarkers for Predicting Kidney Function and Histological Damage in Transplanted Deceased Donor Kidney Recipients

**DOI:** 10.3390/jcm14041349

**Published:** 2025-02-18

**Authors:** Andrejus Bura, Gintare Stonciute-Balniene, Audra Banisauskaite, Laura Velickiene, Inga Arune Bumblyte, Antanas Jankauskas, Ruta Vaiciuniene

**Affiliations:** 1 Nephrology Department, Lithuanian University of Health Sciences, 44307 Kaunas, Lithuania; 2 Radiology Department, Lithuanian University of Health Sciences, 44307 Kaunas, Lithuania

**Keywords:** chronic allograft nephropathy, IF/TA (interstitial fibrosis/tubular atrophy), kidney transplantation, MRI (magnetic resonance imaging), non-invasive biomarkers

## Abstract

**Background/Objectives**: Kidney transplantation (kTx) is the preferred treatment for end-stage kidney disease. Limited evaluation of structural changes in transplanted kidneys hinders the timely prediction of disease progression and the implementation of treatment modifications. Protocol biopsies provide valuable insights but are invasive and carry risks of biopsy-related complications. This study investigates whether multiparametric magnetic resonance imaging (MRI), including T1 and T2 mapping and diffusion-weighted imaging (DWI), can predict kidney function and the progression of interstitial fibrosis and tubular atrophy (IF/TA) in the early post-transplant period. **Methods**: A prospective study was conducted at The Hospital of Lithuanian University of Health Sciences Kauno Klinikos from May 2022 to March 2024. Thirty-four patients receiving kidney transplants from deceased donors underwent baseline biopsies and post-transplant MRI scans. Follow-up assessments included kidney function evaluation, biopsies, and MRI scans at three months post-transplant. **Results**: Significant correlations were observed between MRI parameters and kidney function: T1 and apparent diffusion coefficient (ADC) corticomedullary differentiation (CMD) correlated with eGFR at discharge (r = −0.338, *p* = 0.05; r = 0.392, *p* = 0.022, respectively). Linear and logistic regression models demonstrated that post-transplant T1 and ADC CMD values significantly predicted kidney function at discharge. Furthermore, T1 CMD values measured 10–15 days post-transplant predicted IF/TA progression at three months post-kTx, with an area under the curve of 0.802 (95% CI: 0.616–0.987, *p* = 0.001) and an optimal cut-off value of −149.71 ms. The sensitivity and specificity were 0.818 and 0.273, respectively (Youden’s index = 0.545). T2 mapping was not predictive. **Conclusions**: This study highlights the potential immediate clinical utility of MRI-derived biomarkers, particularly ADC and T1 CMD, in centers equipped with advanced imaging capabilities as tools for assessing kidney function in the early post-transplant period. With an AUROC of 0.802, T1 CMD demonstrates strong discriminatory power for predicting IF/TA progression early in the post-transplant period.

## 1. Introduction

Kidney transplantation (kTx) is the optimal treatment for end-stage chronic kidney disease (CKD) [1]. However, it involves numerous proinflammatory and profibrotic processes interacting with structural and functional changes in transplanted kidneys. Standard tests, such as estimated glomerular filtration rate (eGFR) and albuminuria, have been criticized for reflecting only the consequences of disease progression rather than identifying early changes. Novel diagnostic methods enabling earlier detection of chronic alterations in transplanted kidneys represent an unmet clinical need.

Promising results have been shown in proteomics, metabolomics, RNA biomarkers, and cell-free DNA studies. Unfortunately, these biomarkers are still undergoing validation and are not yet widely used in clinical practice [2]. In kTx, markers such as donor-derived cell-free DNA and urinary CXCL10 have shown promise in detecting rejection and monitoring graft health. These markers offer the potential to complement traditional measures like serum creatinine [3]. The Kidney Donor Risk Index, which predicts post-transplant outcomes by assessing multiple donor and transplant characteristics, does not incorporate histological or other methods to evaluate structural changes in transplanted kidneys [4]. To address this gap, some centers perform protocol needle biopsies [5]. Findings evaluated using the Banff classification [6] provide valuable insights into subclinical rejection, calcineurin inhibitor toxicity, and chronic graft fibrosis, aiding in predicting graft survival and informing decisions to prolong kidney function [7]. However, kidney biopsies are time-consuming and carry a low but potential complication rate of gross hematuria, bleeding requiring transfusion and major complication were 3.18% [95% confidence interval (95% CI), 2.31–4.19], 0.31% (95% CI, 0.15–0.52) and 0.89% (95% CI, 0.61–1.22) [8].

Chronic allograft nephropathy, histologically defined as interstitial fibrosis and tubular atrophy (IF/TA) of unknown etiology [7], poses a significant challenge kTx recipients. Given these challenges, imaging techniques such as MRI offer a promising complementary tool for non-invasive kTx structure and function assessment. While MRI does not replace graft biopsy—the gold standard for diagnosing graft dysfunction—it provides valuable additional information, particularly when combined with clinical findings, to better understand ongoing pathological processes in transplanted kidneys. The European Union COST Action PARENCHIMA working groups have aimed to integrate multiparametric magnetic resonance imaging (MRI) of kidneys into clinical practice [9]. Advances in MRI enable the collection of multiple quantitative measures to assess kidney morphology, tissue microstructure, oxygenation, blood flow, and perfusion within a single scan session. Studies using MRI to evaluate structural and functional changes in kTx have shown promising predictive values for subsequent graft function decline [10]. For example, the apparent diffusion coefficient (ADC) has demonstrated higher sensitivity to fibrosis in kTx than eGFR [11]. T1 and ADC corticomedullary differentiation (CMD) correlated with eGFR and fibrosis in animal studies [12], and ADC CMD was identified as an independent predictor of kidney function decline in the study by Berchtold et al. [13]. According to the Echeverria-Chasco R et al. [14], diffusion-weighted imaging (DWI) and T1 mapping have shown diagnostic and prognostic potential in kidney transplantation, providing insights into allograft microstructure and functional changes. These MRI techniques offer advantages over traditional measures, enabling early detection of delayed graft function and predicting graft outcomes. Due to the scarcity of publications addressing ongoing structural changes in kTx during the early and intermediate post-transplantation periods, we conducted a prospective clinical study involving patients transplanted with kidneys from brain-dead donors. We evaluated protocol biopsies of kTx at time-zero and three months post-transplantation, correlating these data with structural MRI measures, including T1 mapping, T2 mapping, and ADC. These MRI parameters provide insights into renal microstructure and function. This study aims to elucidate the potential of MRI biomarkers to predict early kTx function and structural changes.

## 2. Materials and Methods

### 2.1. Study Design

This observational, prospective cohort study included recipients of deceased-donor kTx at The Hospital of Lithuanian University of Health Sciences Kauno Klinikos between May 2022 and March 2024. Ethical approval (22 February 2022, Nr. BE-2-12) was obtained from the City Region Biomedical Ethics Committee, and informed consent was secured from all participants. Postperfusion biopsies (zero-time biopsies) were performed on all patients during kTx. MRI scans were conducted 10–15 days post-transplantation following a 10-day isolation period required due to immunosuppression induction. During the follow-up period, protocol biopsies were conducted three months post-kTx, with MRI scans performed one day before the protocol biopsy. Kidney biopsies were evaluated by a pathologist using the Banff 2022 classification [6].

### 2.2. Study Population

During the study period, 40 cadaveric kTx were performed. Recipients experiencing primary non-function (PNF; n = 1) or claustrophobia (n = 5) were excluded from the analysis. PNF was defined as a permanent lack of graft function from the time of transplantation. Claustrophobia is a fear of enclosed spaces (claustro means closed), according to D.M. Hudson et al. [15] Research occurrence is low, but it makes a cost impact and possibly could implement non-quality imaging. The initial analysis included 34 patients, with 30 patients remaining in the study at the 3-month follow-up (4 patients withdrew for social reasons). Standard immunosuppressive induction therapy was administered pre-transplant, consisting of methylprednisolone, mycophenolate mofetil, and either Basiliximab or anti-thymocyte globulin in high immunological risk cases (Table 1). Deceased donor kidneys were preserved using either the cold storage immersion method or the LifePort kidney transporter machine. Data were collected on recipients’ demographic and clinical characteristics, donors’ clinical characteristics, and graft function during the early post-transplant phase and up to three months post-transplantation.

### 2.3. Evaluation of Graft Function

Delayed graft function (DGF) was defined as the requirement for hemodialysis (HD) within the first week after transplantation [16]. However, this definition has limitations, such as misclassification if a single HD session is required within the first hours post-transplant due to hyperkalemia or hyperhydration [17]. To evaluate early graft function, we applied the definition proposed by Isaac E. Hall and colleagues [18], categorizing graft function as immediate (IGF), slow (SGF), or delayed (DGF). Serum creatinine (Scr) reduction was calculated as the difference between Scr at 0 h and Scr on day 7, divided by Scr at 0 h. SGF was defined as an Scr reduction ratio < 0.7 without HD, while IGF was defined as a ratio ≥ 0.7. DGF required at least one HD session within seven days, excluding cases of early postoperative hyperkalemia or hypervolemia.

Among the participants, 20 had IGF, 3 had SGF, and 11 had DGF. Due to the small number of SGF and DGF cases, these groups were merged for the final analysis. Patients were further categorized based on eGFR at discharge and eGFR at the three-month follow-up:

- eGFR ≥ 60 mL/min/1.73 m^2^ (n = 17) vs. < 60 mL/min/1.73 m^2^ (n = 17).
- eGFR ≥ 60 mL/min/1.73 m^2^ (n = 11) vs. < 60 mL/min/1.73 m^2^ (n = 19).

### 2.4. MRI Biomarkers

Magnetic resonance relaxometry (MRR) is feasible, offering highly reproducible pixel-wise parametric maps of tissue-specific T1 and T2 relaxation times. These maps can be utilized for the non-invasive assessment of parenchymal changes associated with kidney injury and graft dysfunction. The ADC measures the diffusion of water molecules within tissues, offering valuable insights into tissue structure and function. Together, MRR and diffusion imaging serve as powerful tools for predicting the severity of interstitial fibrosis in kidneys [19,20].

### 2.5. Imaging Protocol

All examinations were performed using a 3 Tesla clinical scanner (MAGNETOM Skyra, Siemens Medical Solutions, Erlangen, Germany) with a 32-channel body coil for abdominal coverage, following published consensus recommendations [21,22]. Localization: HASTE sequences were used to locate the transplanted kidney. T1 and T2 Mapping: Three coronal slices (8 mm thickness) through the ventral, middle, and dorsal regions of the kTx were acquired. T1 mapping utilized a modified Look–Locker inversion recovery (MOLLI) scheme with a single-shot balanced steady-state free precession (SSFP) sequence. T2 mapping used a T2-weighted single-shot balanced SSFP sequence. T1- and T2-weighted images underwent motion correction. Inline motion correction was achieved by acquiring MOLLI images on a frame-by-frame basis. The motion of each pixel within the image was tracked and adjusted, enabling precise compensation for specific motion patterns.

The diffusion-weighted imaging (DWI): Axial DWI was performed using a free-breathing echo-planar technique with 4 b-values: 0, 100, 200 and 800 s/mm^2^ (Table A1).

### 2.6. Imaging Analysis

The raw MRI images were post-processed and analyzed using parametric MRI software (https://www.parametricmri.com/, Philadelphia, PA, USA, V1.0). ADC maps were generated by fitting the DWI signal across all b-values using a mono-exponential model. Quantitative maps reflecting renal tissue characteristics (T1 and T2 mapping [23]) were also analyzed.

**Figure 1:**
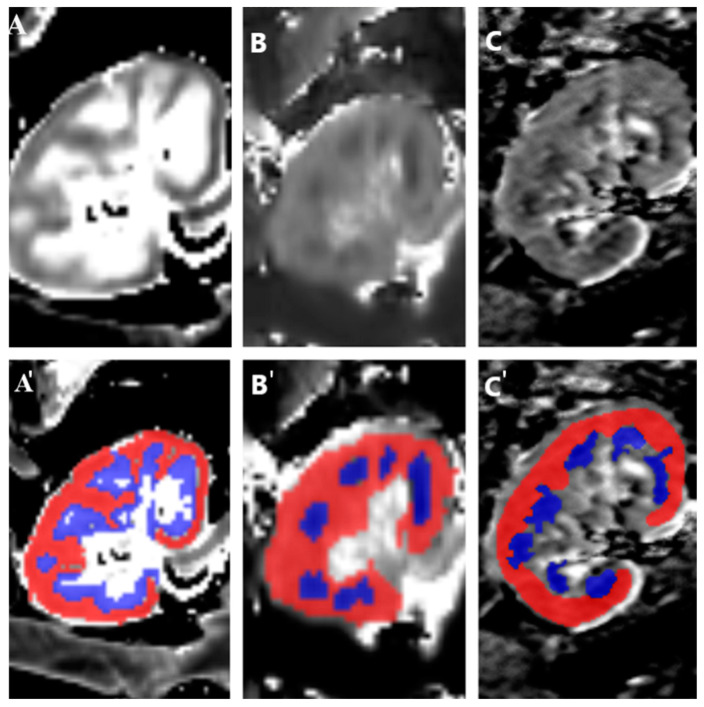
The post-processing images of transplanted kidneys: T1 map (**A**), T2 map (**B**), and ADC map (**C**). Segmentation of renal MRI data. The cortex and medulla were identified map, and ROIs were semi-automatically delineated in the cortex (red) and medulla (blue) on the T1 map (**A’**), T2 map (**B’**), and ADC map (**C’**).

Two observers, a radiologist and a nephrologist, performed image analyses independently (Appendix B). Regions of interest (ROIs) for the cortex and medulla were delineated, ensuring uniform size and location for each region (Figure 1). Measurements were averaged across observers, and the mean and standard deviations for the cortex and medulla were calculated. Corticomedullary ratios (CMD) were computed to highlight relative differences between cortical and medullary values and normalize the data.

### 2.7. Kidney Transplant Biopsy Evaluation

The main structural changes were assessed by the IF/TA score and progression using Banff scores for interstitial fibrosis (ci) and tubular atrophy (ct). Banff acute injury scores were excluded from both time-zero and three-month follow-up protocol biopsies due to the absence of acute rejection signs (Appendix C).

Based on IF/TA status, kidneys were categorized at time-zero as follows:

- No or minimal IF/TA (n = 28).
- Mild IF/TA (n = 5).

At three months post-transplantation, the groups were reclassified:

- No or minimal IF/TA (n = 11).
- Mild-to-moderate IF/TA (n = 11).

### 2.8. Statistical Analyses

Statistical analyses were performed using IBM SPSS Statistics, version 29.0.2.0 (IBM Corp., Armonk, NY, USA). Continuous variables were expressed as mean (standard deviation, SD) or median (interquartile range, IQR). Categorical variables were reported as counts and percentages. The mean square contingency coefficient was used to assess associations between binary variables. Comparisons of continuous variables (T_1_ map, T_2_ map, ADC and clinical data) were made using the Student’s *t*-test for normally distributed data or the Mann–Whitney U-test for non-normally distributed data. Categorical variables (clinical data) were analyzed using the Chi-square test or Fisher’s exact test. Associations between continuous variables were assessed using Pearson’s correlation coefficient for normally distributed data or Spearman’s rank correlation coefficient for non-normally distributed data. Univariate linear regression evaluated the impact of MRI variables on eGFR at discharge. Multivariable linear regression (stepwise selection) identified independent predictors of graft function at discharge, with results reported as regression coefficients (B) and 95% confidence intervals (CI). Multiple logistic regression (backward selection) assessed the relationship between biomarkers and eGFR ≥ 60 mL/min/1.73 m^2^ at discharge, with results reported as odds ratios (OR), 95% CI, and *p*-values derived from the likelihood ratio test. Receiver operating characteristic (ROC) curve analyses were conducted to assess the ability of T1 CMD 10–15 days after kTx to predict IF/TA at three months post-kTx. Optimal cut-off points were determined using the Youden J index. Interobserver variability between the radiologist and nephrologist was assessed using the intraclass correlation coefficient (ICC), with an ICC > 0.75 indicating good reliability (Appendix B). All statistical tests were two-sided, with significance defined as *p* < 0.05. The graphics were created using Jeffreys’s Amazing Statistics software, version 0.19.2.

## 3. Results

### 3.1. Patient Characteristics and Clinical Findings in the Early Post-Transplant Period

A total of 34 patients were enrolled in the study during the early post-KTx period. Among these, 20 recipients had IGF, while 14 had slow or delayed graft function (SGF + DGF). A comparison of recipient and donor characteristics between the two groups is presented in Table 1. Notably, the IGF group had a significantly shorter cold ischemic time for kidney preservation.

### 3.2. Kidney Function and Structural MRI Values in the Early and Intermediate Post-Transplant Period

Structural MRI performed at 10–15 days and three months post-kTx revealed no significant differences between the IGF and SGF + DGF groups (Table 2). However, the T1 CMD values at 10–15 days were significantly better in patients with eGFR ≥ 60 mL/min/1.73 m^2^ at discharge (−164.09 ± 62.44 ms vs. −117.00 ± 66.23 ms, *p* = 0.041). This trend persisted three months post-kTx, with T1 CMD remaining better in the same group (−191.47 ± 47.60 ms vs. −138.68 ± 35.70 ms, *p* = 0.002). No other MRI sequences demonstrated significant differences at these time points. Additionally, ADC CMD values at three months post-kTx were higher in patients with preserved kidney function (106.54 ± 38.4 vs. 61.53 ± 62.46, *p* = 0.04). Other MRI sequences showed no significant differences between groups (Table A3).

**Figure 2:**
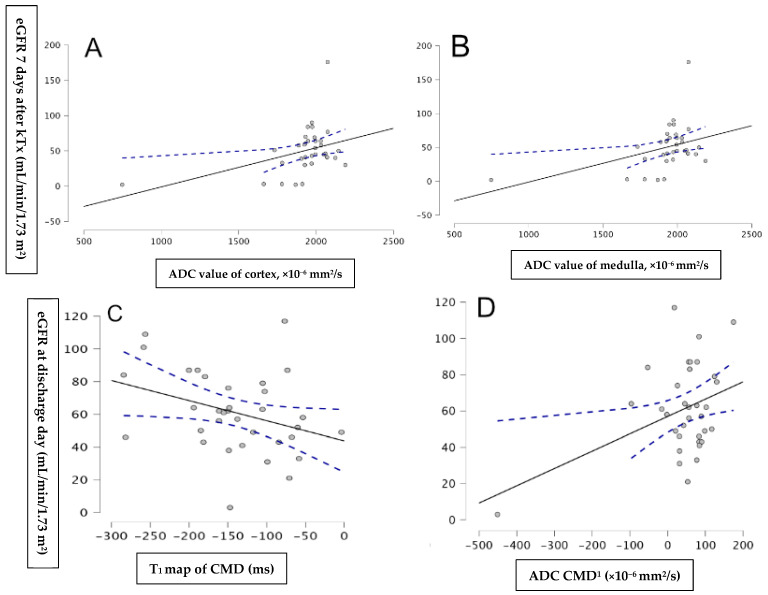
Correlation between eGFR 7 days after kTx and ADC cortical values (**A**), ADC medulla values (**B**), T1 map CMD (**C**), ADC CMD (**D**), and eGFR at discharge day.

Correlation analysis revealed a positive relationship between eGFR at 7 days post-kTx and both ADC cortex (r = 0.401, *p* = 0.019) and ADC medulla values (r = 0.517, *p* = 0.002). A significant Pearson correlation was also observed between eGFR at discharge and both T1 CMD (r = −0.338, *p* = 0.05) and ADC CMD (r = 0.392, *p* = 0.022) (Figure 2).

### 3.3. Histological Parameters and MRI Structural Changes: At Time-Zero Biopsy

**Figure 3:**
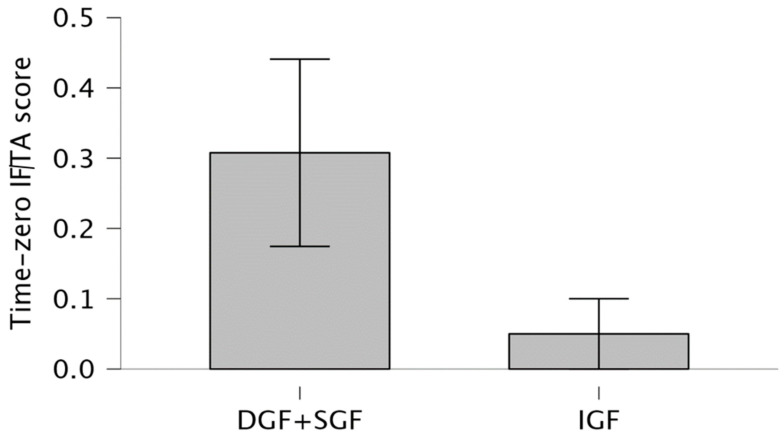
Difference in IF/TA scores between study groups. SGF+DGF group 0.308 (0.48) than in IGF 0.05 (0.224) (*p* = 0.045).

The SGF + DGF group showed higher IF/TA scores compared with the IGF group (0.308 ± 0.48 vs. 0.05 ± 0.224, *p* = 0.045) (Figure 3).

**Figure 4:**
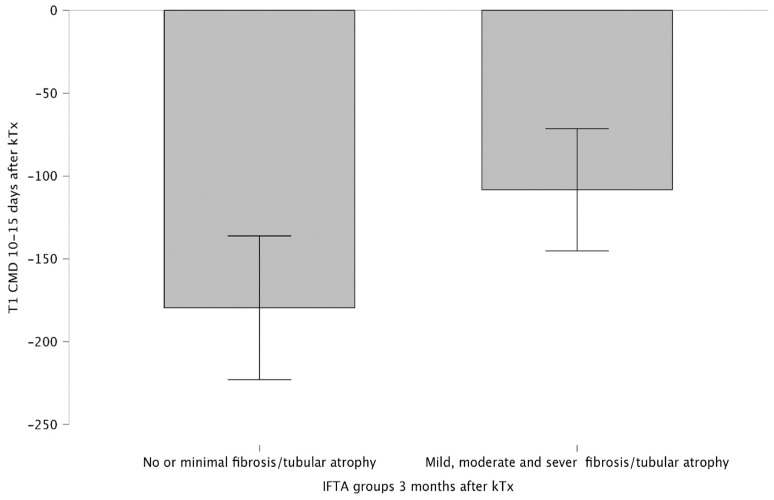
MRI T1 CMD 10–15 days after kTx difference between IF/TA groups 3 months after kTx.

Between IF/TA groups at time-zero biopsy, there was no significant relation with MRI T1, T2 maps, and ADC values 10–15 days and 3 months post-kTx (Table A4). However, T1 CMD values of 10–15 days after kTx were significantly higher in the no/minimal IF/TA group (n = 11) compared to the mild/moderate IF/TA group (n = 11) 3 months post-kTx biopsies, at −179.56 ± 64.59 and −108.34 ± 54.88 ms, respectively (*p* = 0.011) (Figure 4, Table A5).

Statistical analysis showed no significant correlation between MRI T1, T2 maps, or ADC values at 10–15 days post-kTx and Banff variables at time-zero or three-month biopsies (Table A6). Similarly, there was no correlation between MRI structural parameters at three months post-kTx and IF/TA scores at either biopsy time point (Table A7).

### 3.4. Predictive Performance of MRI Biomarkers for Early Graft Function

Univariate linear regression analysis revealed a significant negative association between T1 CMD at 10–15 days and eGFR at discharge (β = −0.126, 95% CI [−0.240 to −0.013], *p* = 0.03). Similarly, ADC CMD showed a positive association with eGFR at discharge (β = 0.095, 95% CI [0.015–0.176], *p* = 0.022). These associations remained significant in multivariable linear regression after adjusting for cold ischemic time. (Table 3).

Logistic regression analysis incorporating T1 CMD, ADC CMD, and cold ischemic time confirmed T1 CMD and cold ischemic time as independent predictors of eGFR ≥ 60 mL/min/1.73 m^2^ at discharge (Table 4).

### 3.5. The Prognostic Performance of T1 CMD in Predicting IF/TA Condition

**Figure 5:**
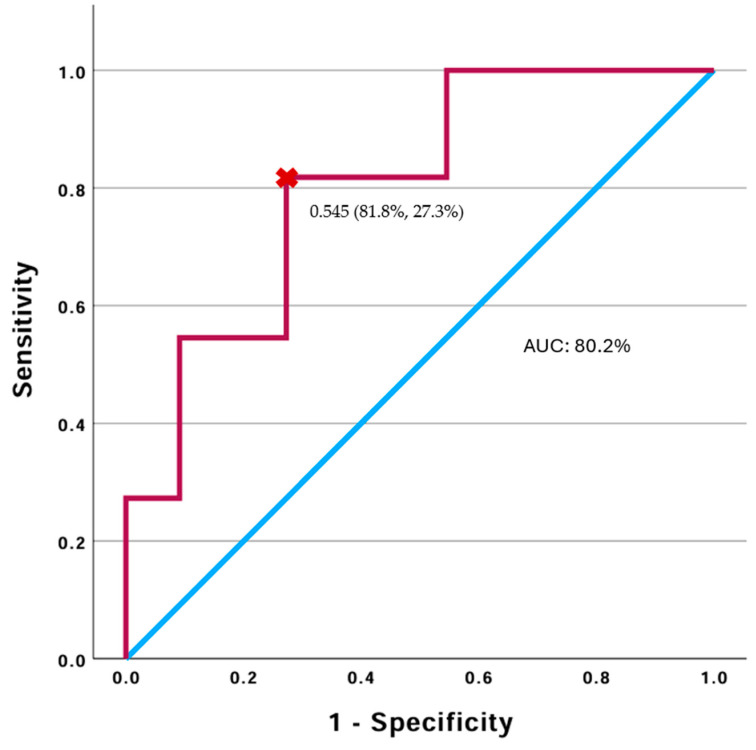
The diagnostic accuracy of the receiver-operating characteristic curve of T1 CMD (red line) for predicting IF/TA progression 3 months after kTx.

ROC analysis demonstrated that T1 CMD values at 10–15 days post-kTx predicted IF/TA progression at three months. The area under the ROC curve (AUROC) for T1 CMD was 0.802 (95% CI: 0.616–0.987, *p* = 0.001), with an optimal cut-off value of −149.71 ms. The sensitivity and specificity were 0.818 and 0.273 (Youden’s index = 0.545) (Figure 5).

## 4. Discussion

The general conclusion of this study is that significant correlations between eGFR and the MRI parameters ADC and T1 suggest their potential as biomarkers for kidney function in the early and intermediate post-transplant periods. Early MRI T1 mapping post-transplantation could be particularly useful in predicting IF/TA progression three months after kTx.

This study has several notable strengths. First, the patient selection was highly focused. The study included recipients without any acute pathological findings at the time of the zero biopsy, and none of the patients experienced acute rejection or tubular necrosis during the study period. This homogeneity allowed us to isolate the effect of MRI parameters on kidney function and IF/TA progression without the confounding influence of other pathological conditions. Consistent monitoring enabled a clear evaluation of the natural progression of IF/TA during the study period. However, we recognize that this selection criterion may limit the generalizability of our findings. In clinical reality, early post-transplant complications such as rejection, acute tubular necrosis, or infectious complications often occur and can contribute to IF/TA development through inflammatory mechanisms. The accuracy of T1 CMD in predicting IF/TA progression in a broader cohort—including patients with early acute complications—remains an open question that requires further investigation. Second, the study methodology is comparable to that used by Hueper K. [24], who performed kidney biopsies in a DGF cohort. However, while Hueper K.’s study identified histopathological changes such as rejection, acute tubular necrosis, and glomerulosclerosis, these pathologies were absent in our cohort. This distinction underscores the unique contributions of our study, where the absence of acute pathological conditions allowed for a focused analysis of MRI parameters and their predictive value for IF/TA. Other studies [25,26,27] have reported fewer promising results, particularly regarding reduced T1 CMD in kTx recipients with allograft rejection or acute tubular necrosis. In contrast, our study highlights the predictive utility of T1 CMD when acute pathologies are excluded. The ROC analysis suggests that T1 CMD measured 10–15 days post-kTx is a valuable predictor of chronic allograft nephropathy progression three months later. This indicates that T1 CMD could serve as a non-invasive biomarker to identify high-risk patients, potentially guiding early interventions and monitoring strategies. Chao-Gang Wei et al. [28] have demonstrated that reduced T1 CMD values are associated with fibrosis severity in CKD patients. Consistent with Friedli I. et al.’s [12] findings, we observed a correlation between T1 CMD in the early post-transplant period and GFR at discharge.

In cases of impaired kidney function during the first three months post-kTx, we observed lower ADC values in the cortex, medulla, and CMD, consistent with findings from other studies [10,29,30,31]. Similarly, like other authors [12,32,33,34,35], we found correlations between T1 and ADC parameters and eGFR at seven days and discharge. As observed by Sułkowska et al. [36], there is a trend of reduced ADC values in impaired grafts. Consistent with [14], our study revealed significant differences in T1 CMD among patient groups at 3 months post-kTx, stratified by eGFR levels. Furthermore, T1 CMD exhibited a similar correlation pattern with eGFR. Neither ADC nor T1 mapping effectively differentiated between structural kidney changes in the IGF and DGF+SGF groups. In contrast, renal blood flow sequences showed superior performance in classifying these groups. To our knowledge, human in vivo measurements of renal T2 remain relatively scarce. Like other studies [32,37], we found prolonged T2 relaxation times. However, these values did not correlate with histological data.

Unlike the studies by Friedli I. et al. [38] and Jiang B. et al. [33], we did not observe correlations between MRI sequences and interstitial fibrosis either in the early post-transplant period or three months later. Lisa C. Adams et al. [32] demonstrated that T1 cortical values significantly predict interstitial fibrosis. Other studies using native T1 mapping of patients’ kidneys with chronic kidney disease show a good correlation with renal function and, importantly, in detecting renal fibrosis [28,39]. While MRI is valuable for assessing structural changes in kTx, it does not provide the level of detail offered by histopathological analysis of biopsies. To address this gap, we need improved imaging sequences to make MRI results more comparable to biopsy findings. Our analysis does not clarify the specific mechanisms behind IF/TA progression. Future studies with histopathological validation, inflammatory markers, and a diverse patient population could provide valuable insights into IF/TA and its treatment implications. One of the challenges in achieving consistent analysis across studies is the variation in MRI sequences, which necessitates standardization. The European Union COST Action PARENCHIMA has provided strategic recommendations for future clinical trials involving renal MRI [40]. Despite these recommendations, some smaller studies continue to yield contradictory results. Another challenge lies in the variability of histopathological interpretations of kidney interstitial fibrosis.

This study has several limitations. The small sample size of 34 patients, with even smaller subgroups, limits the generalizability of the findings. Larger, multicenter studies are needed to validate these results and establish more definitive conclusions. In contrast to the findings reported in [41], our study did not find a significant correlation between the degree of interstitial fibrosis and T1 mapping or ADC. This gap suggests that while T1 CMD provides valuable insights, it may not fully reflect the complex pathophysiological changes underlying IF/TA progression. Another limitation of our study was the inability to conduct renal blood oxygenation level-dependent (BOLD) MRI and arterial spin labeling (ASL) MRI. These advanced imaging techniques could have considerable potential for early differentiation between IGF and DGF + SGF as well as for predicting recovery time.

The study’s findings serve as an important starting point for future research. While T1 CMD offers a non-invasive tool with good sensitivity for predicting IF/TA progression, its low specificity and lack of histological correlation highlight areas for further exploration. It underscores the potential of MRI-based biomarkers for early diagnostic and prognostic purposes in kidney transplantation but emphasizes the need for more robust, large-scale investigations to fully elucidate their clinical applicability. Despite these limitations, T1 CMD can serve as a non-invasive tool to stratify patients and implement preventive measures or closer monitoring for those at higher risk of IF/TA progression. Incorporating T1 CMD into post-transplant management could allow clinicians to personalize treatment plans, such as adjusting immunosuppressive regimens or increasing the frequency of imaging studies. The relatively low specificity (27.3%) underscores the need for combining T1 CMD with other biomarkers or clinical indicators to reduce false-positive rates and ensure efficient resource utilization.

In conclusion, the clinical utility of T1 CMD lies in its ability to provide early, non-invasive prediction of adverse outcomes, which can help optimize post-transplant care strategies. However, its use should be supplemented by comprehensive clinical judgment and additional diagnostics to confirm findings. Standardization of MRI sequences, as recommended by the European Union COST Action PARENCHIMA, remains crucial for consistent analysis across studies.

## 5. Conclusions

This study highlights the potential immediate clinical utility of MRI-derived biomarkers, particularly ADC and T1 CMD, in centers equipped with advanced imaging capabilities as tools for assessing kidney function in the early post-transplant period. With an AUROC of 0.802, T1 CMD demonstrates strong discriminatory power for predicting IF/TA progression early in the post-transplant period. The high sensitivity (0.818) of this metric makes it a valuable tool for identifying at-risk patients, enabling proactive adjustments to immunosuppressive therapy, closer monitoring, or timely interventions to mitigate graft damage. The study’s limitations, particularly the small sample size and lack of correlation with Banff scores, highlight the need for larger, multicenter trials to validate these findings. Future research should focus on exploring additional MRI parameters, establishing standardized protocols, and evaluating long-term outcomes.

## Figures and Tables

**Table 1:** Relationship between demographic and clinical data after kidney transplantation.

Recipients	IGF ^1^	SGF + DGF ^1^	*p*
	n = 20	n = 14	
Gender male (%)	15 (44.1)	10 (29.4)	0.816
Age (years)	45.2(15.22)	44.64 (11.09)	0.279
Duration of kidney replacement therapy (months)	28.5 (5–51.75)	13 (13–50.75)	0.148
HLA mismatch	3 (1–3)	3 (1–3)	0.457
Kidney disease (%)	Chronic glomerulonephritis: 5 cases (25%) Diabetic nephropathy: 1 case (5%) Autosomal dominant polycystic kidney disease: 4 cases (20%) Hypertensive nephropathy: 1 case (5%) Other: 9 cases (45%)	Chronic glomerulonephritis: 1 case (7.1%) Diabetic nephropathy: 1 case (7.1%) Autosomal dominant polycystic kidney disease: 2 cases (14.3%) Hypertensive nephropathy: 2 cases (14.3%) Other: 8 cases (57.1%)	
Immunossuppressive regimen	Methylprednisolone: 100% Mycophenolate mofetil: 100% Tacrolimus: 100% Induction therapy: Anti-thymocyte globulin: 1 case (5%) INN-basiliximab: 19 cases (95%)	Methylprednisolone: 100% Mycophenolate mofetil: 100% Tacrolimus: 100% Induction therapy: Anti-thymocyte globulin: 2 cases (14.3%) INN-basiliximab: 12 cases (85.7%)	
Creatinine before kTx (μmol/L)	756 (606.25–1100)	678 (420.5–923.5)	0.416
eGFR 3 days after kTx (mL/min/1.73 m^2^)	72.6 (31.55)	19.5 (18.25)	<0.001
eGFR 7 days after kTx (mL/min/1.73 m^2^)	63.5 (31.99)	31.43 (23.89)	0.003
eGFR at discharge day (mL/min/1.73 m^2^)	67.35 (21.65)	52.07 (26.62)	0.08
eGFR at 3 months post kTx (mL/min/1.73 m^2^)	61.36 (18.35)	53.04 (23.28)	0.260
Donors			
Age (years)	47.65 (15.93)	54.36 (10.43)	0.178
Expanded criteria donor ^2^ (%)	7 (35)	7 (50)	0.382
Cold ischemic time of transplanted kidney (min)	717.85 (193.68)	919.14 (240.28)	0.011

^1^ Determination of IGF, SGF, and DGF was performed using the following formula: the difference between serum creatinine (Scr) at 0 h and Scr on day 7 divided by Scr at 0 h. In those who did not require HD, SGF was defined as a creatinine reduction ratio less than 0.7, and IGF was defined as a ratio greater than or equal to 0.7. DGF was defined by at least one HD session within 7 days of transplant. ^2^ Expanded criteria donor—60 years old and more or more than 50 years old with two criteria: arterial hypertension, serum creatinine > 130 μmol/L, death underwent cerebral vascular damage. Data are given as a number (%), mean (SD) or median (IQR). Refer to the evaluation of graft function for the patient classification.

**Table 2:** Comparison of magnetic resonance imaging data between study groups based on early graft function.

	IGF	SGF + DGF	*p*
**Recipients**	**n = 20**	**n = 14**	
**Strucural MRI data 10–15 days after kTx**			
T_1_ map of cortex (ms)	1619.95 (119.72)	1498.52 (289.57)	0.157
T_1_ map of medulla (ms)	1767.35 (118.23)	1629.28 (321.13)	0.144
T_1_ map of CMD ^1^ (ms)	−147.40 (70.44)	−130.76 (64.97)	0.489
T_2_ map of cortex (ms)	78.71 (6.31)	79.72 (17.27)	0.818
T_2_ map of the medulla (ms)	78.95 (7.06)	78.57 (13.88)	0.921
T_2_ map of CMD ^1^ (ms)	0.23 (4.40)	1.15 (5.78)	0.456
ADC value of cortex (×10^−6^ mm^2^/s)	1970.10 (98.85)	1866.71 (348.04)	0.297
ADC value of medulla (×10^−6^ mm^2^/s)	1925.10 (81.30)	1826.89 (220.32)	0.131
ADC CMD ^1^ (×10^−6^ mm^2^/s)	45.00 (60.08)	39.82 (144.15)	0.886
**Structural MRI 3 months after kTx**			
**Recipients**	**n = 18**	**n = 12**	
T_1_ map of cortex (ms)	1536.57 (129.72)	1575.66 (131.68)	0.428
T_1_ map of medulla (ms)	1706.17 (156.15)	1716.35 (116.53)	0.849
T_1_ map of CMD ^1^ (ms)	−169.60 (46.62)	−140.69 (44.76)	0.102
T_2_ map of cortex (ms)	78.62 (6.42)	80.56 (8.82)	0.490
T_2_ map of the medulla (ms)	75.31 (5.28)	77.18 (7.66)	0.434
T_2_ map of CMD ^1^ (ms)	3.38 (2.46)	3.31 (2.68)	0.941
ADC value of cortex (×10^−6^ mm^2^/s)	2003.04 (132.30)	1963.25 (111.17)	0.381
ADC value of medulla (×10^−6^ mm^2^/s)	1905.45 (105.03)	1898.25 (86.01)	0.838
ADC CMD ^1^ (×10^−6^ mm^2^/s)	97.58 (54.69)	65.00 (58.79)	0.138

^1^ Corticomedullary differentiation (CMD) is based on the following formula: cortical value − medulla value. Data given mean (SD). See Section 2.3 for patient classification.

**Table 3:** Multivariate linear regression for evaluation of factors relevant to eGFR on discharge day.

Model	β Coefficient, 95% Confidence Interval (CI)	*p*
1 unadjusted analysis		
T1 CMD ^1^ 10–15 days after kTx	−0.126 (−0.240 to −0.013)	0.030
2 unadjusted analysis		
ADC CMD ^1^ 10–15 days after kTx	0.095 (0.015 to 0.176)	0.022
3 adjusted analysis		
T1 CMD ^1^ 10–15 days after kTx	−0.132 (−0.242 to −0.022)	0.021
ADC CMD ^1^ 10–15 days after kTx	0.087 (0.013 to 0.162)	0.023
Cold ischemic time (minutes)	−0.028 (−0.060 to 0.005)	0.091

^1^ Corticomedullary differentiation (CMD) is based on the following formula: cortical value − medulla value.

**Table 4:** Multivariate logistic regression for evaluation of factors relevant to eGFR ≥ 60 mL/min/1.73 m^2^ on discharge day.

Model	Odds Ratio, 95% Confidence Interval (CI)	*p*
T1 CMD ^1^ 10–15 days after kTx	−0.016 (0.970 to 0.999)	0.032
ADC CMD ^1^ 10–15 days after kTx	0.003 (0.989 to 1.017)	0.646
Cold ischemic time (minutes)	−0.005 (0.991 to 1.000)	0.033

^1^ Corticomedullary differentiation (CMD) is based on the following formula: cortical value − medulla value.

## Data Availability

The data presented in this study are available upon request from the corresponding author. They are not publicly available due to limited ethical approval.

## Abbreviations

The following abbreviations are used in this manuscript:

kTx	Kidney transplantation
CKD	Chronic kidney disease
IF/TA	Interstitial fibrosis and tubular atrophy
MRI	Magnetic resonance imaging
ADC	The apparent diffusion coefficient
eGFR	Estimated glomerular filtration rate
PNF	Primary non-function
DGF	Delayed graft function
HD	Hemodialysis
IGF	Immediate graft function
SGF	Slow graft function
Scr	Serum creatinine
MRR	Magnetic resonance relaxometry
MOLLI	A modified Look–Locker inversion recovery
SSFP	A single-shot balanced steady-state free precession
DWI	Diffusion-weighted imaging
CMD	Corticomedullary differentiation
SD	Standard deviation
IQR	Interquartile range
B	Regression coefficients
95% CI	95% confidence intervals
OR	odds ratios
ROC	Receiver operating characteristic
ICC	The intraclass correlation coefficient

## Appendix A

**Table A1:** Imaging modalities and corresponding parameters.

	T1 MAP	T2 MAP	DWI
Sequence t.	GRE/separate	GRE/separate	EPI/separate
Orientation	Coronal	Coronal	Transversal
Res. control	Breath-hold	Breath-hold	-
RT (ms)	357.73	294.63	5800
ET (ms)	1.01	1.22	61
IT (ms)	211	-	-
Voxel (mm^3^)	2.0 × 2.0 × 8.0	2.6 × 2.6 × 8.0	1.0 × 1.0 × 5.0
FOV (mm^2^)	500 × 500	500 × 500	380 × 380
Flip ang. (deg)	35	12	-
Slices	3	3	35
EPI fac.	-	-	113
Fat suppression	Off	Off	SPAIR
Acceleration fac.	2 (GRAPPA)	2 (GRAPPA)	2 (GRAPPA)
Scan time (min)	0:45	0:39	3:40

(-): not applicable, RT: repetition time, ET: echo time, IT: inversion time, GRE: gradient echo sequences, EPI: echo planar imaging, GRAPPA: gene-realized autocalibrating partially acquisitions, SPAIR: spectral attenuated inversion recovery.

## Appendix B

### Imagine Analyses

A radiologist and a nephrologist performed the image analyses. They evaluated T1, T2, and apparent diffusion coefficient (ADC) values in the middle section of the transplanted kidney in the coronal view. Each transplanted kidney was conditionally divided into three regions: upper, middle, and lower parts. For each patient, 8 regions of interest (ROIs) were drawn in these three parts for each kidney, targeting the cortex and medulla (Figure 1). The ROIs for the cortex and medulla had uniform sizes per region.

For T1 and T2 maps, which were acquired in the oblique coronal plane, ROIs were copied from the T1 maps into the corresponding slices in the T2 maps. Manual adjustments were made where regions between the T1 and T2 maps did not match. For ADC maps acquired in the axial plane, ROIs were manually adjusted on the cortical and medullary parts of the transplanted kidneys.

The intraclass correlation coefficients (ICCs) between two independent specialists for the kidney transplants (kTx) were as follows, measured 10–15 days post-transplantation:

- T1 map, cortical: 0.889 (95% CI 0.778–0.945); medulla: 0.906 (95% CI 0.814–0.953) (*p* < 0.001)
- T2 map, cortical: 0.976 (95% CI 0.949–0.989); medulla: 0.910 (95% CI 0.806–0.958) (*p* < 0.001)
- ADC, cortical: 0.921 (95% CI 0.826–0.962); medulla: 0.829 (95% CI 0.659–0.914) (*p* < 0.001)

At the three-month follow-up, the ICCs were:

- T1 map, cortical: 0.880 (95% CI 0.748–0.943); medulla: 0.855 (95% CI 0.696–0.931) (*p* < 0.001)
- T2 map, cortical: 0.954 (95% CI 0.904–0.978); medulla: 0.916 (95% CI 0.824–0.960) (*p* < 0.001)
- ADC, cortical: 0.860 (95% CI 0.706–0.933); medulla: 0.822 (95% CI 0.626–0.915) (*p* < 0.001)

## Appendix C

### KTx Biopsy Evaluation

Time-zero biopsies were performed on 34 patients; however, one biopsy was excluded due to an inadequate sample. During the follow-up period, 22 protocol biopsies were performed. One patient was contraindicated due to anticoagulant use, two patients had recurrent urinary tract infections, and five patients declined to undergo a kidney biopsy. All biopsy evaluations were conducted at the National Center of Pathology. None of the patients experienced acute rejection following kidney transplantation.

**Table A2:** Biopsy data.

	Time-Zero kTx Biopsy		3 Months Post-kTx Biopsy	
	Mean (SD)	N (Total/Pathology)	Mean (SD)	N (Total/Pathology)
Glomerular count	13.15 (8.47)	33/33	24.64 (11.93)	22/22
Glomerular sclerosis, score	0.56 (0.94)	33/12	1.14 (1.91)	22/11
Glomerulitis, score	0.09 (0.29)	33/3	0.23 (0.53)	22/4
Inflamantory infiltrates	0.0 (0.0)	33/0	0.0 (0.0)	22/0
Peritubulat capillaritis, score	0.0 (0.0)	33/0	0.05 (0.21)	22/1
Interstitial fibrosis, score	0.12 (0.33)	33/4	0.36 (0.58)	22/7
Tubular atrophy, score	0.03 (0.17)	33/1	0.5 (0.51)	22/11
Arteriolar hyalinosis, score	0.0 (0.0)	33/0	0.18 (0.39)	22/4
IF/TA* score	1.15 (0.364)	33/5	1.82 (0.91)	22/11

Due to the poor representativeness of the median (IQR), we report only the mean (SD) to provide a more comprehensive understanding of the distribution. IF/TA*—interstitial fibrosis and tubular atrophy.

## Appendix D

**Table A3:** Comparison of magnetic resonance imaging data between study groups based on discharge and intermediate graft function.

	eGFR ≥ 60 mL/min/1.73 m^2^	eGFR < 60 mL/min/1.73 m^2^	*p*
Recipients	n = 17	n = 17	
**Strucural MRI data 10–15 days after kTx**			
T_1_ map of cortex (ms)	1594.28 (139.47)	1545.62 (268.38)	0.512
T_1_ map of medulla (ms)	1758.37 (149.10)	1662.62 (288.27)	0.236
T_1_ map of CMD ^1^ (ms)	−164.09 (62.44)	−117.00 (66.23)	0.041
T_2_ map of cortex (ms)	81.95 (8.42)	76.07 (13.82)	0.160
T_2_ map of the medulla (ms)	80.66 (8.26)	76.82 (11.59)	0.295
T_2_ map of CMD ^1^ (ms)	1.29 (1.76)	−0.75 (6.82)	0.278
ADC value of cortex (×10^−6^ mm^2^/s)	1980.5 (108.62)	1874.56 (312.67)	0.196
ADC value of medulla (×10^−6^ mm^2^/s)	1926.24 (99.76)	1843.08 (196.54)	0.130
ADC CMD ^1^ (×10^−6^ mm^2^/s)	54.26 (66.44)	31.47 (128.43)	0.520
**Structural MRI 3 months after kTx**			
	**eGFR ≥ 60 mL/min/1.73 m^2^**	**eGFR < 60 mL/min/1.73 m^2^**	
**Recipients**	**n = 11**	**n = 19**	
T_1_ map of cortex (ms)	1569.44 (98.76)	1542.23 (146.27)	0.589
T_1_ map of medulla (ms)	1760.91 (101.02)	1680.91 (152.38)	0.132
T_1_ map of CMD ^1^ (ms)	−191.47 (47.60)	−138.68 (35.70)	0.002
T_2_ map of cortex (ms)	79.38 (5.83)	79.39 (8.31)	0.997
T_2_ map of the medulla (ms)	76.04 (5.87)	76.07 (6.67)	0.989
T_2_ map of CMD ^1^ (ms)	3.35 (1.96)	3.33 (2.89)	0.981
ADC value of cortex (×10^−6^ mm^2^/s)	2034.59 (94.61)	1947.08 (122.74)	0.051
ADC value of medulla (×10^−6^ mm^2^/s)	1928.05 (78.43)	1885.55 (98.15)	0.231
ADC CMD ^1^ (×10^−6^ mm^2^/s)	106.55 (38.65)	61.52 (62.46)	0.040

^1^ Corticomedullary differentiation (CMD) is based on the following formula: cortical value − medulla value. Data are given as mean (SD).

## Appendix E

**Table A4:** Comparison of magnetic resonance imaging data between study groups divided by time-zero biopsy IF/TA.

Recipients	No or Minimal Fibrosis/Tubular Atrophy	Mild Fibrosis/Tubular Atrophy	*p*
	n = 28	n = 5	
**Strucural MRI data 10–15 days after kTx**			
T_1_ map of cortex (ms)	1596.21 (1504.39–1662.33)	1731.59 (1618.40–1803.55)	0.247
T_1_ map of medulla (ms)	1756.48 (1588.75–1830.30)	1881.54 (1723.73–1953.02)	0.364
T_1_ map of CMD ^1^ (ms)	−142.44 (−181.92–(−)82.37)	−149.47 (−149.95–(−)105.34)	0.942
T_2_ map of cortex (ms)	79.02 (74.58–82.48)	82.43 (80.78–86.30)	0.123
T_2_ map of the medulla (ms)	77.72 (73.25–83.58)	82.99 (81.89–85.29)	0.157
T_2_ map of CMD ^1^ (ms)	0.67 (−0.934–3.04)	1.25 (0.10–1.61)	0.976
ADC value of cortex (×10^−6^ mm^2^/s)	1971.75 (1920–2036.88)	1990.5 (1931–1996)	0.782
ADC value of medulla (×10^−6^ mm^2^/s)	1907.5 (1862.38–1979.25)	1888.5 (1854–1919)	0.609
ADC CMD ^1^ (×10^−6^ mm^2^/s)	54.5 (24.75–83)	102 (77–117)	0.060
**Structural MRI 3 months after kTx**			
T_1_ map of cortex (ms)	1564.73 (1464.34–1609.65)	1621.20 (1618.54–1678.91)	0.101
T_1_ map of medulla (ms)	1726.45 (1644.41–1751.37)	1753.16 (1749.11–1792.94)	0.201
T_1_ map of CMD ^1^ (ms)	−168.52 (−191.01–(−)138.48)	−129.25 (−134.62–(−)127.92)	0.114
T_2_ map of cortex (ms)	77.01 (74.64–82.75)	78.95 (78.83–80.74)	0.323
T_2_ map of the medulla (ms)	75.09 (72.88–80.23)	74.83 (72.90–76.09)	0.978
T_2_ map of CMD ^1^ (ms)	2.94 (1.73–3.69)	5.94 (5.91–7.31)	0.053
ADC value of cortex (×10^−6^ mm^2^/s)	1951.50 (1900.13–2047.13)	2004 (1958–2031.5)	0.436
ADC value of medulla (×10^−6^ mm^2^/s)	1895.50 (1837.5–1943.38)	1876 (1874.5–1923.5)	0.758
ADC CMD ^1^ (×10^−6^ mm^2^/s)	81.75 (47–115.25)	108 (83.5–114.5)	0.544

^1^ Corticomedullary differentiation (CMD) is based on the following formula: cortical value − medulla value. Data are given as median (IQR).

## Appendix F

**Table A5:** Comparison of magnetic resonance imaging data between study groups divided 3 months post-kTx biopsy IF/TA.

Recipients	No or Minmal Fibrosis/Tubular Atrophy	Mild Fibrosis/Tubular Atrophy	*p*
	n = 11	n = 11	
**Strucural MRI data 10–15 days after kTx**			
T_1_ map of cortex (ms)	1624.84 (49.33)	1552.44 (213.29)	0.478
T_1_ map of medulla (ms)	1804.40 (85.95)	1660.78 (251.33)	0.171
T_1_ map of CMD ^1^ (ms)	−179.56 (64.59)	−108.34 (54.88)	0.016
T_2_ map of cortex (ms)	80.36 (7.65)	81.97 (9.52)	0.492
T_2_ map of the medulla (ms)	79.83 (8.13)	80.19 (7.72)	0.545
T_2_ map of CMD ^1^ (ms)	0.53 (3.53)	1.77 (5.41)	0.717
ADC value of cortex (×10^−6^ mm^2^/s)	1997.73 (70.46)	1928.86 (27.64)	0.200
ADC value of medulla (×10^−6^ mm^2^/s)	1932.23 (55.77)	1869.64 (137.4)	0.151
ADC CMD ^1^ (×10^−6^ mm^2^/s)	65.50 (49.54)	59.23 (61.87)	0.844
**Structural MRI 3 months after kTx**			
T_1_ map of cortex (ms)	1533.71 (139.90)	1568.08 (126.07)	0.365
T_1_ map of medulla (ms)	1687.76 (170.38)	1721.02 (126.67)	0.217
T_1_ map of CMD ^1^ (ms)	−154.05 (54.21)	−152.94 (35.46)	0.797
T_2_ map of cortex (ms)	78.75 (6.89)	82.78 (8.20)	0.217
T_2_ map of the medulla (ms)	75.34 (5.52)	78.88 (7.03)	0.217
T_2_ map of CMD ^1^ (ms)	3.4 (3.38)	3.9 (2.37)	0.797
ADC value of cortex (×10^−6^ mm^2^/s)	1993.86 (105.86)	1987.05 (134.76)	0.898
ADC value of medulla (×10^−6^ mm^2^/s)	1908.05 (90.63)	1919.36 (103.03)	0.699
ADC CMD ^1^ (×10^−6^ mm^2^/s)	85.82 (40.62)	67.68 (73.41)	0.694

^1^ Corticomedullary differentiation (CMD) is based on the following formula: cortical value − medulla value. Data are given as mean (SD).

## Appendix G

**Table A6:** Spearman‘s correlations of magnetic resonance imaging and time zero biopsy data.

	IF/TA Score	Interstitial Fibrosis (ci)	Tubular Atrophy (ct)
**Strucural MRI data 10–15 days after kTx**			
T_1_ map of cortex (ms)	0.213 (*p* = 0.234)	0.146 (*p* = 0.417)	0.167 (*p* = 0.353)
T_1_ map of medulla (ms)	0.169 (*p* = 0.348)	0.098 (*p* = 0.589)	0.167 (*p* = 0.353)
T_1_ map of CMD ^1^ (ms)	0.018 (*p* = 0.922)	0.059 (*p* = 0.746)	−0.074 (*p* = 0.681)
T_2_ map of cortex (ms)	0.295 (*p* = 0.114)	0.302 (*p* = 0.105)	0.054 (*p* = 0.778)
T_2_ map of the medulla (ms)	0.272 (*p* = 0.146)	0.302 (*p* = 0.105)	0.011 (*p* = 0.955)
T_2_ map of CMD ^1^ (ms)	−0.011 (*p* = 0.953)	−0.019 (*p* = 0.920)	0.011 (*p* = 0.955)
ADC value of cortex (×10^−6^ mm^2^/s)	0.053 (*p* = 0.768)	0.029 (*p* = 0.872)	0.056 (*p* = 0.758)
ADC value of medulla (×10^−6^ mm^2^/s)	−0.098 (*p* = 0.589)	−0.088 (*p* = 0.627)	−0.037 (*p* = 0.837)
ADC CMD ^1^ (×10^−6^ mm^2^/s)	0.417 (*p* = 0.056)	0.341 (*p* = 0.052)	0.223 (*p* = 0.213)
**Structural MRI 3 months after kTx**			
T_1_ map of cortex (ms)	0.316 (*p* = 0.094)	0.215 (*p* = 0.262)	0.248 (*p* = 0.194)
T_1_ map of medulla (ms)	0.251 (*p* = 0.189)	0.143 (*p* = 0.458)	0.248 (*p* = 0.194)
T_1_ map of CMD ^1^ (ms)	0.306 (*p* = 0.107)	0.239 (*p* = 0.212)	0.181 (*p* = 0.348)
T_2_ map of cortex (ms)	0.196 (*p* = 0.307)	0.215 (*p* = 0.262)	0 (*p* = 1)
T_2_ map of the medulla (ms)	−0.011 (*p* = 0.955)	0.072 (*p* = 0.712)	−0.158 (*p* = 0.413)
T_2_ map of CMD ^1^ (ms)	0.426 (*p* = 0.051)	0.335 (*p* = 0.076)	0.248 (*p* = 0.194)
ADC value of cortex (×10^−6^ mm^2^/s)	0.153 (*p* = 0.429)	0.275 (*p* = 0.149)	−0.203 (*p* = 0.290)
ADC value of medulla (×10^−6^ mm^2^/s)	0.065 (*p* = 0.736)	0.084 (*p* = 0.666)	−0.203 (*p* = 0.907)
ADC CMD ^1^ (×10^−6^ mm^2^/s)	0.120 (*p* = 0.535)	0.263 (*p* = 0.168)	−0.248 (*p* = 0.194)

^1^ Corticomedullary differentiation (CMD) is based on the following formula: cortical value − medulla value.

## Appendix H

**Table A7:** Spearman’s correlations of magnetic resonance imaging and 3 months post-kTx biopsy data.

	IF/TA Score	Interstitial Fibrosis (ci)	Tubular Atrophy (ct)
Structural MRI 3 Months After kTx			
T_1_ map of cortex (ms)	0.219 (*p* = 0.328)	0.131 (*p* = 0.561)	0.208 (*p* = 0.353)
T_1_ map of medulla (ms)	0.313 (*p* = 0.157)	0.238 (*p* = 0.287)	0.279 (*p* = 0.208)
T_1_ map of CMD ^1^ (ms)	0.052 (*p* = 0.819)	0.029 (*p* = 0.898)	0.064 (*p* = 0.776)
T_2_ map of cortex (ms)	0.224 (*p* = 0.315)	0.058 (*p* = 0.797)	0.279 (*p* = 0.208)
T_2_ map of the medulla (ms)	0.218 (*p* = 0.330)	0.073 (*p* = 0.748)	0.279 (*p* = 0.208)
T_2_ map of CMD ^1^ (ms)	0.092 (*p* = 0.682)	0.063 (*p* = 0.780)	0.064 (*p* = 0.776)
ADC value of cortex (×10^−6^ mm^2^/s)	0.115 (*p* = 0.611)	0.146 (*p* = 0.518)	0.036 (*p* = 0.874)
ADC value of medulla (×10^−6^ mm^2^/s)	0.084 (*p* = 0.711)	0.0 (*p* = 0.1)	0.093 (*p* = 0.680)
ADC CMD ^1^ (×10^−6^ mm^2^/s)	−0.023 (*p* = 0.920)	0.034 (*p* = 0.881)	−0.093 (*p* = 0.680)

^1^ Corticomedullary differentiation (CMD) is based on the following formula: cortical value − medulla value.
